# Integration of Evolutionary Features for the Identification of Functionally Important Residues in Major Facilitator Superfamily Transporters

**DOI:** 10.1371/journal.pcbi.1000522

**Published:** 2009-10-02

**Authors:** Jouhyun Jeon, Jae-Seong Yang, Sanguk Kim

**Affiliations:** 1 Division of Molecular and Life Science, Pohang University of Science and Technology, Pohang, Korea; 2 School of Interdisciplinary Bioscience and Bioengineering, Pohang University of Science and Technology, Pohang, Korea; National Cancer Institute, United States of America and Tel Aviv University, Israel

## Abstract

The identification of functionally important residues is an important challenge for understanding the molecular mechanisms of proteins. Membrane protein transporters operate two-state allosteric conformational changes using functionally important cooperative residues that mediate long-range communication from the substrate binding site to the translocation pathway. In this study, we identified functionally important cooperative residues of membrane protein transporters by integrating sequence conservation and co-evolutionary information. A newly derived evolutionary feature, the co-evolutionary coupling number, was introduced to measure the connectivity of co-evolving residue pairs and was integrated with the sequence conservation score. We tested this method on three Major Facilitator Superfamily (MFS) transporters, LacY, GlpT, and EmrD. MFS transporters are an important family of membrane protein transporters, which utilize diverse substrates, catalyze different modes of transport using unique combinations of functional residues, and have enough characterized functional residues to validate the performance of our method. We found that the conserved cores of evolutionarily coupled residues are involved in specific substrate recognition and translocation of MFS transporters. Furthermore, a subset of the residues forms an interaction network connecting functional sites in the protein structure. We also confirmed that our method is effective on other membrane protein transporters. Our results provide insight into the location of functional residues important for the molecular mechanisms of membrane protein transporters.

## Author Summary

Major Facilitator Superfamily (MFS) transporters are one of the largest families of membrane protein transporters and are ubiquitous to all three kingdoms of life. Structural studies of MFS transporters have revealed that the members of this superfamily share structural homology; however, due to weak sequence similarity, their structural similarity has only been found after structural determination. Even after the structures were solved, painstaking efforts were needed to detect functionally important residues. The identification of functionally important cooperative residues from sequences may provide an alternative way to understanding the function of this important class of proteins. Here, we show that it is possible to identify functionally important residues of MFS transporters by integrating two different evolutionary features, sequence conservation and co-evolutionary information. Our results suggest that the conserved cores of evolutionarily coupled residues are involved in specific substrate recognition and translocation of membrane protein transporters. Also, a subset of the identified residues comprises an interaction network connecting functional sites in the protein structure. The ability to identify functional residues from protein sequences may be helpful for locating potential mutagenesis targets in mechanistic studies of membrane protein transporters.

## Introduction

The identification of functionally important cooperative residue is important for understanding the allosteric pathways of proteins. Cooperative residues are responsible for long-range allosteric communication from the substrate binding sites to the translocation pathways of membrane protein transporters [1]. A number of methods have been proposed for the identification of functionally important residues in proteins. Based on the notion that functionally important residues tend to be conserved within a protein family [2],[3], sequence conservation analyses have been applied to identify specific functional sites, such as substrate/ligand binding residues [4], protein-protein interfaces [5], active sites of enzymes [6], and residues responsible for functional specificity [7]. Meanwhile, co-evolutionary analyses, which were introduced by the observation that functionally important residues are likely to co-evolve with other functional residues to reduce the effects of mutations [8], have been applied to identify energetically and/or evolutionarily coupled interactions between the domains of complex proteins [9], the interaction sites of protein complexes [10], and the allosteric pathways of proteins [11],[12]. One drawback of these approaches is that residues may be conserved or co-evolved due to several underlying causes, such as the maintenance of protein structure, interaction, and folding, as well as functional constraint [13],[14]. Therefore, a method that can quantify and detect functional constraints from the evolutionary information in protein sequences would greatly aid the identification of functionally important residues in proteins [15].

Membrane protein transporters are involved in two-state allosteric communication, which mediates the propagation of regulatory information from the substrate binding site to the translocation pathway through large conformational changes [1]. These conformational changes could be brought about through cooperative residues [16]. Recent studies have suggested that cooperative residues are conserved [17] or evolutionary coupled [18] to maintain allosteric communication. Furthermore, it has been proposed that co-evolved pairs of moderately conserved residues are important for protein function [19]. Thus, it may be possible to combine sequence conservation and co-evolutionary analyses to identify the cooperative residues of membrane protein transporters. To do this, we derived a new method for identifying the cooperative residues of membrane protein transporters by integrating two different evolutionary features. We extracted functional information from multiple evolutionary constraints based on the following deduction: we took advantage of the fact that clusters of cooperative residues might be co-evolutionary connected not only by proximal but also distal residues in order to mediate allosteric communication [18]. When we considered a protein as a co-evolving network of residues, high connectivity described the functional essentiality of a single residue. Based on these, we hypothesized that cooperative residues lining the substrate binding and translocation pathway are likely to be conserved and have more co-evolutionarily coupled partners than non-functional residues, showing high connectivity in a co-evolution network. To test our hypothesis, we introduced a co-evolutionary coupling number (CN) to measure the connectivity of co-evolving residue pairs in a co-evolution network. We then integrated CN with sequence conservation score and investigated the functional roles and structural positions of the conserved cores of co-evolutionarily coupled residues.

We initially applied our method to the MFS transporters, LacY, GlpT, and EmrD, for which crystal structures have been solved and whose functional residues have been characterized well enough to evaluate the performance of our method. MFS transporters represent one of the largest and most diverse superfamily of membrane protein transporters and are ubiquitous to all three kingdoms [20]. The identification of cooperative residues of MFS transporters may be helpful in inferring their allosteric mechanisms, including substrate recognition and translocation. MFS transporters move various substrates (e.g., sugar, drug, metabolites, and anions) in different directions across cell membranes using a unique combination of residues in their transmembrane regions [21]. One MFS transporter, lactose permease (LacY), is a symporter that catalyzes the coupled translocation of lactose and H^+^ [22]. Another, glycerol-3-phosphate transporter (GlpT), mediates the exchange of glycerol-3-phosphate and inorganic phosphate in an antiport manner [23]. Multi-drug transporter, EmrD, is an antiporter that exports a diverse group of chemically unrelated drugs [24]. Using our method, we found that conserved cores of evolutionarily coupled residues comprise residue interaction networks connecting the specific substrate recognition site and translocation pathway of MFS transporters. We also tested our method on other proteins and confirmed that it is effective in identifying the cooperative residues of membrane protein transporters.

## Results

### Evolutionary constraints on the central cavity of MFS transporters

**Figure 1:**
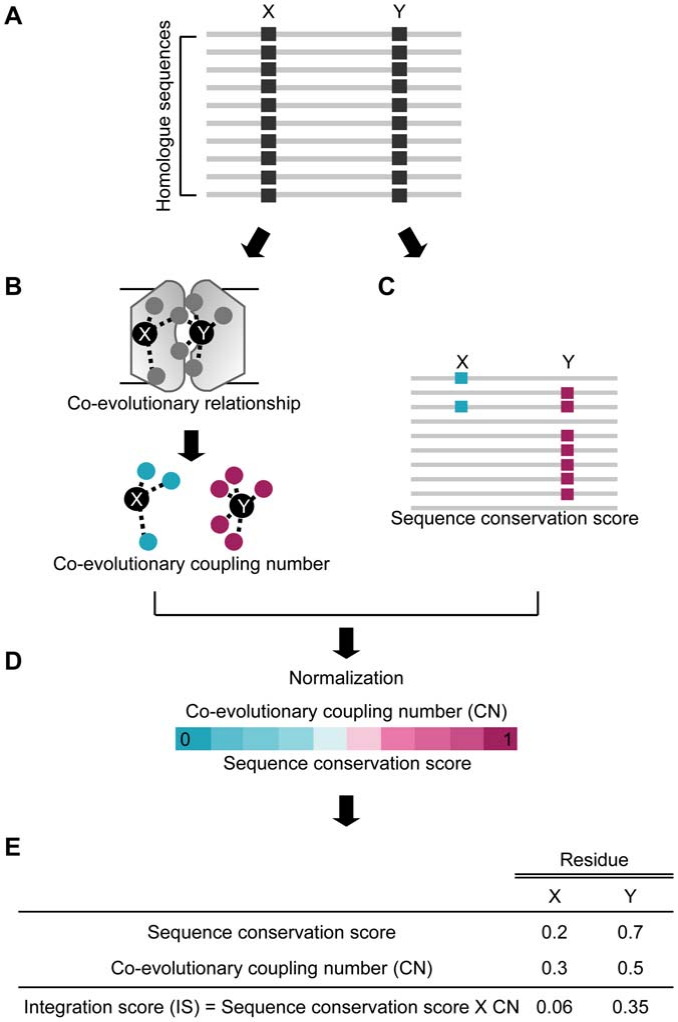
Overview of integrative evolutionary analysis. (A) A schematic view of multiple sequence alignment (MSA) of a protein family. Co-evolution and sequence conservation scores were calculated from homologue sequences. X and Y indicate different residues in a protein. (B) Quantification of the co-evolutionary relationship of a single residue. Co-evolutionary coupling number (CN) was defined by the number of co-evolved residue pairs per residue. A dashed line represents co-evolving residue pairs. Circles represent the co-evolved partners of residues X and Y. (C) Measurement of sequence conservation scores of residues X and Y. Blue and red squares indicate conserved amino acids of residues X and Y, respectively. (D) Normalization of CN and sequence conservation scores by assigning a score raging from 0 to 1. (E) Integration score (IS) was obtained by multiplying CN and sequence conservation score.

We devised a new evolutionary feature, co-evolutionary coupling number (CN), and integrated it with the sequence conservation score to select functionally important cooperative residues from protein sequences. Figure 1 diagrams the proposed method. First, we measured co-evolution and sequence conservation scores from homologue sequences. Second, we formulated the CN by counting the number of co-evolving residue pairs per residue. Finally, we calculated a quantitative integration score (IS) of each residue by multiplying sequence conservation score and CN (see Materials and Methods for details).

To examine whether functionally important cooperative residues tend to be conserved and have many co-evolved partners, we compared average IS, CN, and sequence conservation scores between central cavity residues and non-cavity residues. The central cavity of an MFS transporter is mainly composed of functionally important residues that are involved in substrate recognition and are located in the pathway of substrate transport [25]. We found that central cavity residues were significantly more conserved and had many more co-evolved partners than non-cavity residues, resulting in a high IS (Table S1). The average IS of central cavity residues was 3.1 times higher than that of the non-cavity residues (p-value = 2.31×10^−11^). Statistical significance was determined by Student's t-test comparing IS distributions between central cavity and non-cavity residues. We further examined the sequence conservation scores of central cavity residues to confirm our initial assumption that central cavity residues are conserved and evolutionary coupled. From the sliding-window analysis of conservation scores, we found that central cavity residues slowly evolved rather than being completely conserved (Figure S1). Central cavity residues were enriched between the 75^th^ and 90^th^ percentile of sequence conservation scores. The fraction of central cavity residues was sharply reduced after the 90^th^ percentile of sequence conservation. These results suggest that a slow evolution rate allows central cavity residues to be conserved and co-evolutionarily coupled with other residues [26]. Therefore, the integration of sequence conservation and CN can be used to identify central cavity residues.

**Figure 2:**
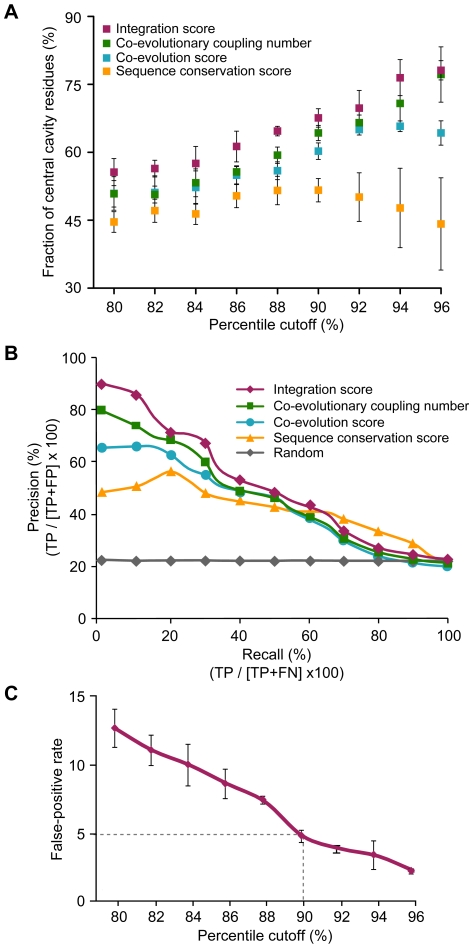
Performance comparisons of three evolutionary features. (A) Fraction of central cavity residues at the given percentile of each evolutionary approach. Red, green, blue, and yellow squares indicate the average fraction of central cavity residues at the given percentile of IS, CN, co-evolution, and sequence conservation scores, respectively. Error bars indicate the standard deviation. (B) Precision-recall curves of four evolutionary approaches. Precision and recall were derived from cavity residues (positive set) and non-cavity residues (negative set) of three MFS transporters. Red, green, blue, and yellow dots represent the average precision of each evolutionary approach in the given recall. (C) Optimization of the percentile cutoff of IS. False-positive rates of IS were shown at the given percentile cutoffs. The dashed line indicates the percentile cutoff of IS with 5% false-positive rate. Error bars indicate the standard deviation between false-positive rates of three different MFS transporters.

To measure the sensitivity of the integrated evolutionary information, we compared our ability to detect central cavity residues by IS, CN, co-evolution, and sequence conservation scores. We examined the fraction of central cavity residues using various percentile cutoffs for IS, CN, co-evolution, and sequence conservation scores. In comparison to the conventional evolutionary approaches, we found IS to be a more effective way to select central cavity residues. As shown in Figure 2A, IS detected 1.1 to 2.2 times more central cavity residues than CN, co-evolution, or sequence conservation score. We also observed that CN had a higher sensitivity for detecting central cavity residues than co-evolution and sequence conservation. This suggests that central cavity residues tend to be co-evolutionarily coupled with many residues rather than being highly conserved.

We compared the precision-recall characteristics of IS, CN, co-evolution, and sequence conservation for a more comprehensive evaluation (*i.e.* how well each of the four approaches do in identifying the central cavity residues). We found that IS was best in the detection of central cavity residues (Figure 2B). Specifically, IS achieved an average precision of 71%, whereas the other evolutionary approaches achieved an average precision of 64% (CN), 58% (co-evolution), and 49% (sequence conservation) at 30% recall. Also, the precision of IS was 3.2-fold higher than a randomly generated set at the same recall. Furthermore, the likelihood ratio of IS was the highest among all four evolutionary approaches (Figure S2). These results indicate that IS can capture the maximum evolutionary property of central cavity residues that would not be apparent by co-evolution or sequence conservation alone.

For the sensitive detection of functional residues, we optimized the percentile cutoff of IS by examining the false-positive rate, which is the fraction of non-cavity residues selected at the given percentile cutoff. We found that, in all three MFS transporters, the 90^th^ percentile of IS discriminated central cavity residues from non-cavity residues with an acceptable false- positive rate of 5% (Figure 2C). Therefore, we used the 90^th^ percentile of IS as a cutoff value to select functional residues for further analyses.

### Identification of the cavity residues of LacY

LacY facilitates the transport of lactose through the inner membrane [22]. LacY is an intensively studied protein of the MFS transporters and its functional residues have been well characterized through mutagenesis [27].

**Figure 3:**
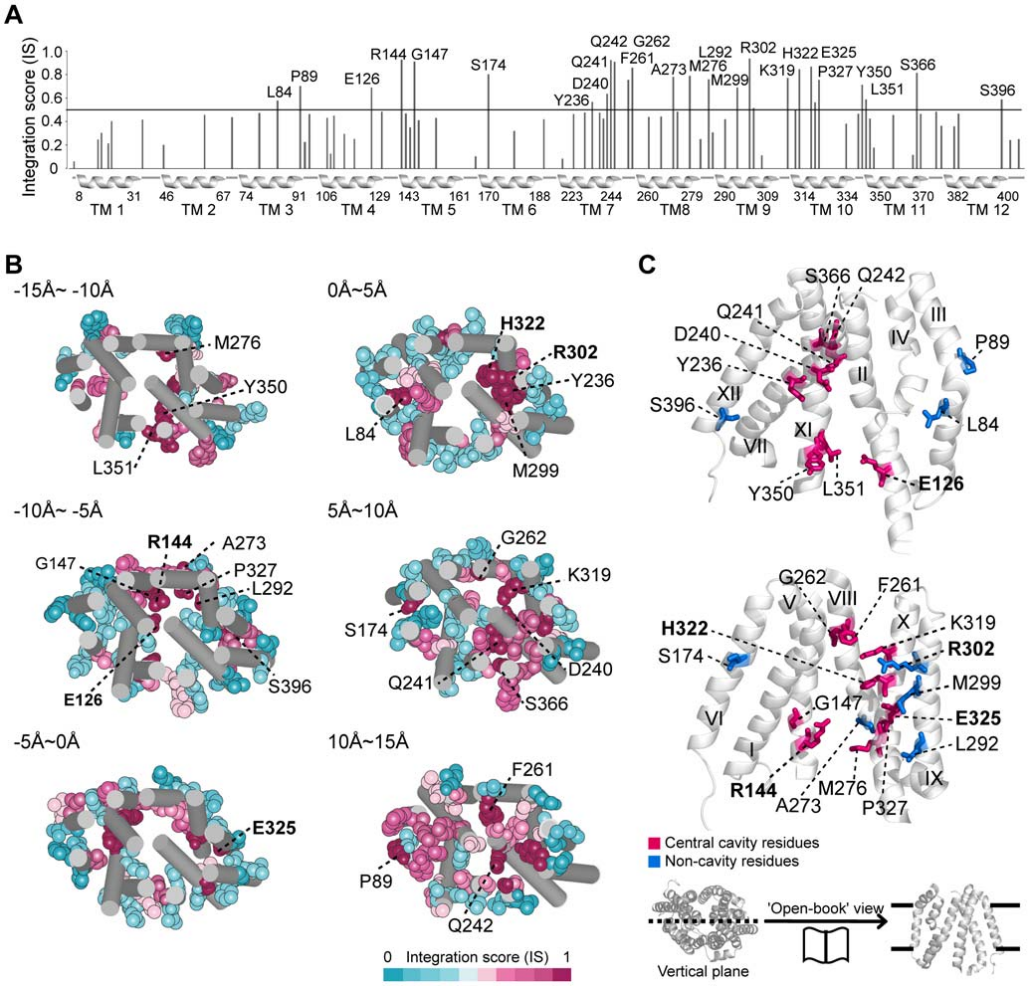
High-IS residues of LacY. (A) IS pattern of LacY. Black line corresponds to the 90^th^ percentile of IS. Transmembrane regions are indicated as helices below the x-axis with boundary residue numbers; 25 detected residues are labeled with residue numbers. (B) Serial sections of LacY structure from cytoplasm (−15Å) to periplasm (15Å). The detected residues are shown as vdW spheres with residue numbers; 5 irreplaceable residues are shown in bold. (C) ‘Open book’ view of the detected residues in LacY. Central cavity and non-cavity residues are shown in red and blue sticks, respectively; five irreplaceable residues are indicated as bold characters. Transmembrane helix numbers are shown in roman numerals.

To investigate whether the high-IS residues are involved in substrate binding and translocation, we identified 25 residues within the 90^th^ percentile of IS (Figure 3A) and found that most residues detected at this cutoff have known functional roles (Table 1). The detected residues were mostly positioned within the substrate translocation pathway of the central cavity (Figure 3B). When we mapped the 25 detected residues on the LacY structure, we found that 17 residues (68% of detected residues) were located in the central cavity (Figure 3C and Table S2). It has been experimentally confirmed that six residues, E126, R144, E269, R302, H322, and E325, are irreplaceable and necessary for LacY operation [27],[28], and we detected five of these residues (Figure 3C, shown in bold). We were able to detect E126, R144, R302, H322, and E325, but missed E269 in the 90^th^ percentile of IS. Meanwhile, the missed residue E269 was found in the 70^th^ percentile of IS.

**Table 1:** Functional implications and experimental evidences of the detected LacY residues.

TM	Position	Residue	Location	Experimentally suggested function	Evidence
3	84	Leu	Non-cavity region	Substrate translocation	[70]
3	89	Pro	Non-cavity region	Not confirmed	-
4	126*	Glu	Central cavity	Substrate binding	[27]
5	144*	Arg	Central cavity	Substrate binding	[27]
5	147	Gly	Central cavity	Lactose accumulation	[28]
6	174	Ser	Non-cavity region	Low expressed	[71]
7	236	Tyr	Central cavity	Substrate translocation	[35]
7	240	Asp	Central cavity	Substrate translocation	[36]
7	241	Gln	Central cavity	Substrate translocation	[72]
7	242	Gln	Central cavity	Substrate translocation	[72]
8	261	Phe	Central cavity	Not confirmed	-
8	262	Gly	Central cavity	Substrate translocation	[34]
8	273	Ala	Non-cavity region	Substrate translocation	[37]
8	276	Met	Central cavity	Substrate translocation	[34]
9	292	Leu	Non-cavity region	Not confirmed	-
9	299	Met	Non-cavity region	Substrate translocation	[37]
9	302*	Arg	Non-cavity region	H^+^ translocation/substrate translocation	[27]
10	319	Lys	Central cavity	Lactose accumulation/substrate translocation	[28]
10	322*	His	Central cavity	H^+^ translocation/substrate translocation	[27]
10	325*	Glu	Central cavity	H^+^ translocation/substrate translocation	[27]
10	327	Pro	Central cavity	Substrate translocation	[33]
11	350	Tyr	Central cavity	Substrate translocation	[28]
11	351	Leu	Central cavity	Not confirmed	-
11	366	Ser	Central cavity	Not expressed	[71]
12	396	Ser	Non-cavity region	Not expressed	[73]

TM represents the transmembrane helix number.

*: indicates the experimentally confirmed irreplaceable residues for LacY operation.

### Residue interaction network is important for the substrate transport mechanism

**Figure 4:**
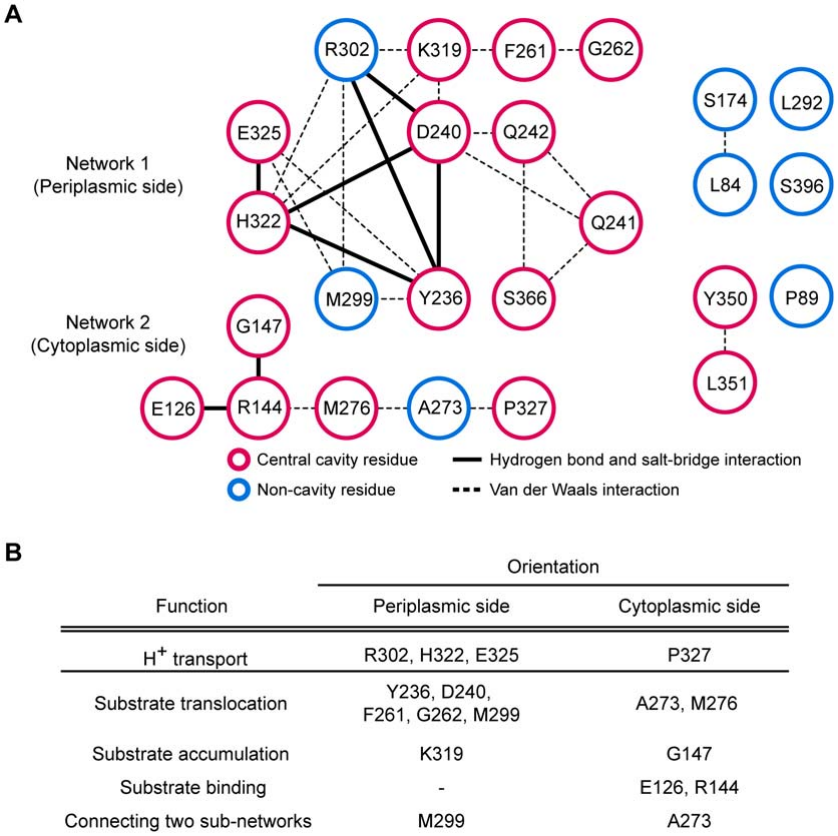
Interaction network of the high-IS residues of LacY. (A) Interaction network of the detected residues in LacY. Eighteen of the detected residues comprised a main interaction network (left), which can be divided into two sub-networks. Red circle represents central cavity residues and blue circle indicates non-cavity residues. Dashed line indicates a van der Waals interaction. Bold line indicates a potential hydrogen bond or salt bridge. (B) Functional implications of the detected residues from the mutational analyses.

Proteins use residue-residue interactions to propagate regulatory information from one functional site to another [29]. We constructed an interaction network by examining the interatomic connectivity among the detected residues. Different types of interactions, such as hydrogen bonds, salt bridges, and van der Waals interactions were assessed by measuring solvent-accessible surface and interatomic distances from the structures of MFS transporters (see Materials and Methods for details). We observed that 23 of the 25 detected residues form an interaction network and 18 of these comprise a main network in the LacY structure (PDB ID: 2CFQ) (Table S3). Of the 18 residues, 15 are central cavity residues known to be essential for LacY operation and 5 of the 18 are irreplaceable (Figure 4A). Hydrogen bonds and salt bridges formed between the residues of Y236, D240, R302, K319, H322, and E325 (bold line in Figure 4A) are known to play important roles in the transduction of the substrate binding signal through the LacY structure [30],[31]. Two irreplaceable residues, E126 and R144, found interact through a hydrogen bond, are involved in substrate binding and release [32].

The functional implications of the interaction network are in accordance with the lactose transport mechanism proposed from LacY mutation experiments [28]. Our main network could be divided into two sub-networks based on orientation: network 1 is located on the periplasmic side and network 2 on the cytoplasmic side (Figure 4A). There is evidence that the residues of both sub-networks simultaneously mediate substrate translocation from opposite sides of the membrane (Figure 4B). Residue E325 detects protonation states and transports H^+^ with R302 and H322 on the periplasmic side, and P327 on the cytoplasmic side [33]. Substrate translocation is mediated by residues Y236, D240, F261, G262, and M299 of network 1 and residues A273 and M276 of network 2 [34]–[36]. Residues K319 in network 1 and G147 in network 2 are involved in substrate accumulation [28]. Among the residues of network 2, E126 and R144 are essential for substrate binding [27]. Residue M299 of network 1 and A273 of network 2 connect two sub-networks and are essential for substrate transport [37]. The functional residues located on both the periplasmic and cytoplasmic sides suggest that the cooperative residues of both networks allow efficient allosteric communication for LacY operation by alternating between two major conformations, inward-facing and outward-facing conformation, respectively [22]. The residues outside the main network, L84, Y350, and L351, lie close to the irreplaceable residue E126 (average Cα distance; 16.5Å) and mediate substrate translocation (Table 1).

### Identification of cavity residues in other MFS transporters

**Figure 5:**
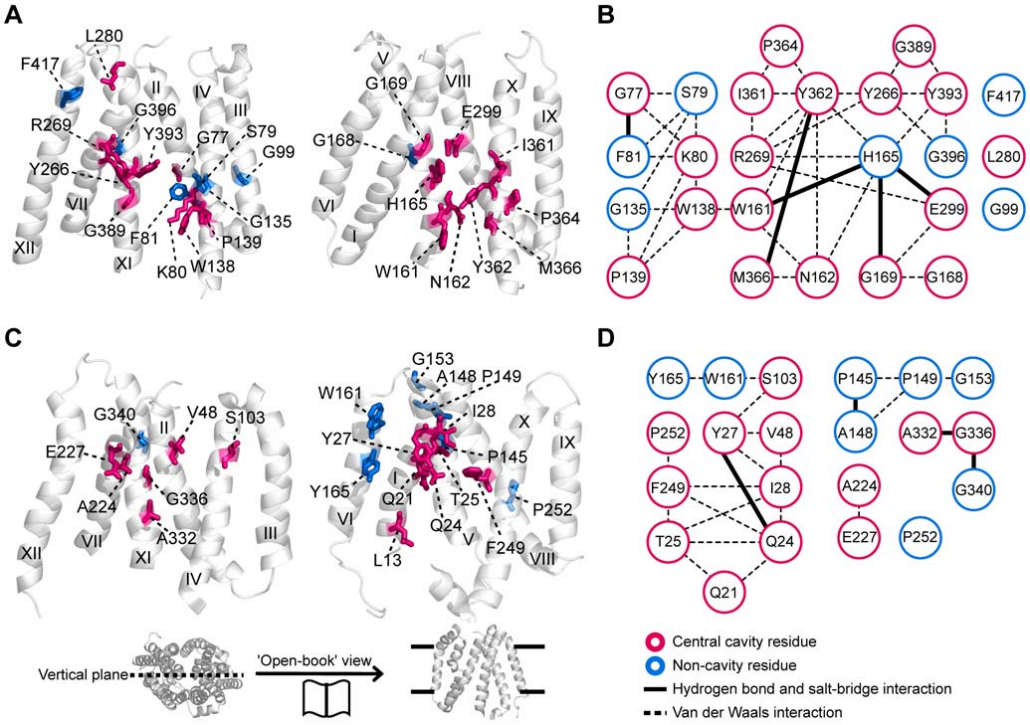
High-IS residues of GlpT and EmrD. (A) ‘Open book’ view of detected residues in GlpT. Central cavity and non-cavity residue are shown in red and blue sticks, respectively. (B) Interaction network of the detected residues in GlpT. Of the 22 network comprising residues (left), 17 residues are found in central cavity (red sticks) and 5 residues are found in the non-cavity region (blue sticks). Dashed line indicates a van der Waals interaction. Bold line indicates a potential hydrogen bond or salt bridge. (C) Mapping high-IS residues onto the EmrD structure. (D) Interaction network of the detected residues of EmrD. Ten residues comprise a main interaction network (left).

The integration of evolutionary features worked well for the identification of functional residues of other family members of MFS transporters. We applied our method to the GlpT and EmrD proteins, the functional residues of which are less well characterized than those of LacY. We found that, similar to LacY, a few residues of GlpT and EmrD have high IS (Figure S3) and they use unique residue combinations for specific substrate binding and translocation. In GlpT, we chose 25 residues within the 90^th^ percentile of IS. When we mapped the residues onto the GlpT structure, we found 18 of 25 residues located along the central cavity (Figure 5A and Table S4). Twenty-two of the detected residues form an interaction network (Figure 5B and Table S5), of which several residues have experimentally confirmed functional roles in substrate binding and translocation (Table S6). For example, residues K80, R269, and H165 have a critical role in substrate binding and residues E299, Y362, and Y393 participate in substrate translocation [23],[38]. In particular, the formation and breakage of salt bridges between residues H165, R269, and E299 are known to involve conformational changes during the transport of glycerol-3-phosphate [39]. Meanwhile, in EmrD, 13 of 21 detected residues are located in the central cavity (Figure 5C and Table S7). Among them, 10 residues comprise the main interaction network associated with H^+^ translocation (Figure 5D and Tables S8, S9). It has been shown that residues Q21, Q24, T25, and I28 are involved in facilitating H^+^ translocation [24]. Compared to LacY and GlpT, little is known about the functional mechanism of EmrD. Our analysis may serve as a guide for future experimental verification of EmrD functional residue location.

### Identification of cavity residues in other membrane protein transporters

**Figure 6:**
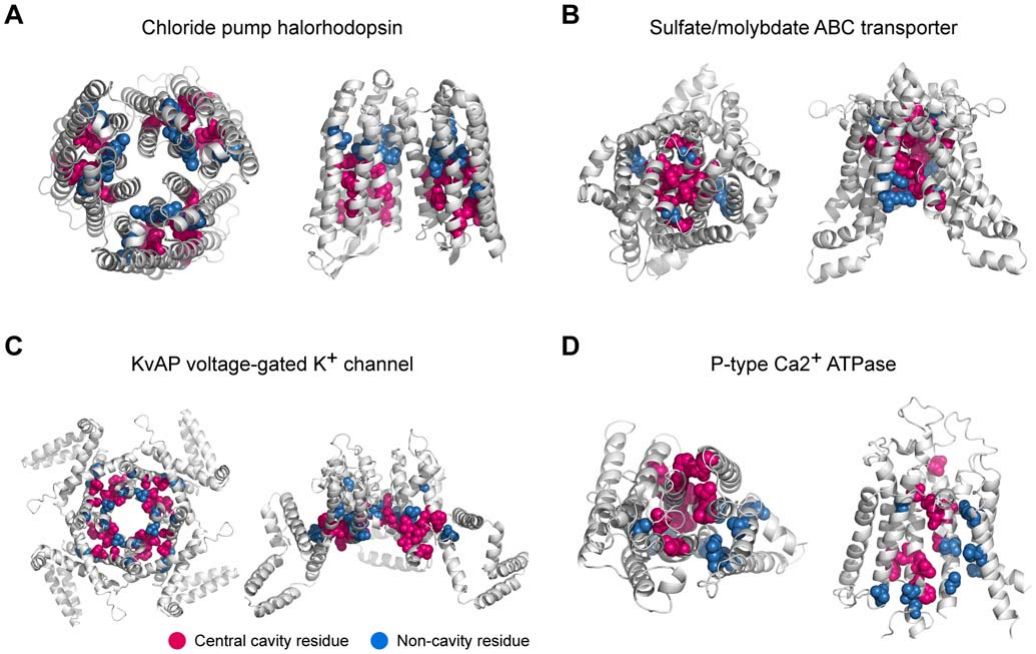
High-IS residues of other membrane protein transporters. Positions of the detected residues are highlighted. Cavity residues are colored red and non-cavity residues are colored blue. The top view (left) and the side view (right) of membrane protein transporters are shown. (A) Chloride pump halorhodopsin (PDB ID: 1E12), (B) Sulfate/molybdate ABC transporter (PDB ID: 3D31), (C) KvAP voltage-gated K^+^ channel (PDB ID: 1ORQ), and (D) P-type Ca2^+^ ATPase (PDB ID: 1WPG).

To ensure that our method works for transporters outside of the MFS superfamily, we tested it on other membrane protein transporters, whose allosteric conformational changes were characterized and whose cavity residues could be selected from crystal structures [40]–[42]. We investigated the positions and annotated functional roles of high-IS residues in 15 membrane protein transporters, such as KvAP and Kv1.2 voltage-gated K^+^ channels, rhodopsin, the chloride pump halorhodopsin, bacteriorhodopsin, sensory rhodopsin, archaerhodopsin, Na^+^/K^+^ ATPase, P-type Ca2^+^ ATPase, plasma membrane ATPase, and the sulfate/molybdate ABC transporter. Membrane protein transporters mediate the movement of ions, solutes, and metabolites across a membrane [43]. We found that, on average, IS selected 2.3 times more cavity residues than random selection (Table 2). Also, we discovered that many high-IS residues were located along the cavity region involved in substrate translocation pathways (Table S10) and comprised interaction networks in the protein structures (Figure S4). For example, in the chloride pump halorhodopsin, 10 of 15 residues were found from the chloride translocation pathway using the 90^th^ percentile of IS (Figure 6A, shown in red spears) [44] and formed an interaction network. In sulfate/molydbate ABC transporter, 9 out of 12 detected residues were located in the substrate translocation pathway (Figure 6B, shown in red spears) [45] and 6 residues comprised an interaction network. In addition, 64% and 55% of the detected residues in the KvAP channel and P-type Ca2+ ATPase were located in the ion conduction pathway and formed an interaction network, respectively (Figures 6C and 6D) [46],[47]. These results showed IS to be an effective way to locate the cavity residues in the tested transporters. Also, in the precision-recall curves of four evolutionary approaches, IS had the highest precision at all levels of recall (Figure S5).

**Table 2:** List of the membrane protein transporters.

Family	PDB	Chain	Protein name	Fraction of cavity residues (%)		Fold-change (%)
				IS*	Random	
MFS	2CFQ	A	Lactose permease	68.00	18.78	362.09.
K^+^ channel	1ORQ	C	KvAP voltage-gated K^+^ Channel	63.64	16.63	382.68
MFS	1PW4	A	Glycerol-3-Phosphate Transporter	72.00	20.88	344.83
ABC transporter	3D31	C	Sulfate/molybdate ABC transporter	75.00	25.48	294.35
MFS	2GFP	A	Multidrug drug transporter	61.90	24.80	249.60
K^+^ channel	2R9R	B	Kv1.2 voltage-gated K^+^ channel	46.15	15.61	295.67
Bacterial rhodopsin	1E12	A	Chloride pump halorhodopsin	66.67	27.22	244.93
ATPase	3B8E	A	Na^+^/K^+^ ATPase	71.43	30.62	233.28
Bacterial rhodopsin	1H2S	A	Sensory rhodopsin 2	41.67	20.48	203.45
Bacterial rhodopsin	2EI4	A	Archaerhodopsin 2	69.23	38.37	180.43
Bacterial rhodopsin	1C3W	A	Bacteriorhodopsin	31.25	20.79	150.31
GPCR	1L9H	A	Rhodopsin	44.44	30.57	145.39
ATPase	1WPG	A	P-type Ca2^+^ ATPase	54.55	36.64	148.88
ATPase	3B8C	A	Plasma membrane ATPase	76.19	57.94	131.50
Bacterial rhodopsin	1UAZ	A	Archaerhodopsin 1	31.25	24.38	128.18

*: The fraction of cavity residues was measured within the 90th percentile of IS.

## Discussion

In this study, we attempted to identify the functionally important cooperative residues of membrane protein transporters from amino acid sequences by integrating two different evolutionary features. We demonstrated that the conserved cores of evolutionarily coupled residues of MFS transporters were mainly located in the substrate translocation pathway. One may question why functionally important residues are conserved and have evolved in a co-dependent manner. It has been suggested that protein sequences may have been robust to environmental and mutational perturbations in the course of evolution in order to preserve protein function [48]. These residues have evolved at a rate that was slow enough to avoid the loss of function [49]. Indeed, we observed that central cavity residues of MFS transporters are moderately conserved and enriched between the 75^th^ and 90^th^ percentile of sequence conservation scores (Figure S1). This slow evolution rate allows correlative substitutions among functional residues, resulting in high co-evolutionary coupling numbers [26].

The presence of an interaction network of cooperative residues is strongly correlated with the pathway of substrate translocation described in other studies [27],[50]. We found that the cluster of cooperative residues comprised an interaction network that may constitute an allosteric pathway connecting the substrate binding site and translocation pathway of MFS transporters. Yifrach and colleagues found that allosteric pathway-lining residues are energetically coupled over long distances and showed that these residues are important for the sequential conformational transition of the Kv channel using electrophysiology recordings techniques [1],[51]. In addition, other researchers have shown that perturbations of conserved residues impair the allosteric communication of protein residues [52],[53]. These results suggest that cooperative residues are evolutionarily coupled and conserved to mediate long-range allosteric communication from the substrate binding site to the translocation pathway of membrane protein transporters.

The efficient regulation of allosteric communication is achieved through the interaction of cooperative residues. Recent network-based structural analyses by Nussinov and colleagues have shown that centrally positioned residues in protein structures maintain the robustness of allosteric pathways through residue-residue interactions [29], [54]. By mapping the detected residues onto the ligand-free (PDB ID: 2CFQ) and ligand-bound (PDB ID: 1PV7) structures, we observed the rearrangement of residue-residue interactions. In particular, irreplaceable substrate binding residues, E126 and R144, had different interatomic contacts between ligand-free and ligand-bound structures (Figure S6). In the ligand-free structure, the guanidine group of R144 forms a salt bridge with the carboxyl group of E126; whereas, in the ligand-bound structure, the two atomic groups directly interact with the substrate by breaking the salt bridge [55],[56]. Also, the rearrangements of hydrogen bonds and salt bridges between residues Y236, D240, R302, K319, H322, and E325 are known to involve conformational changes in LacY [27]. Taken together, we reasoned that the connectivity of the detected residues was changed because efficient conformational changes for substrate transport are regulated by the formation and breakage of interactions between cooperative residues.

We found that some of the high-IS residues in MFS transporters are non-cavity residues, while most of them are positioned in the central cavity to control substrate transport. It may be possible that some of the detected non-cavity residues are also involved in the transport mechanism. For example, it has been reported that a non-cavity residue, R302, of LacY is irreplaceable for substrate transport [27] and connected with central cavity residues, K319, Y236, D240, and H322 (Figure 4B and Table 1). Furthermore, we noticed that some non-cavity residues that have high-IS were found from the residue interaction networks of other membrane protein transporters (Figures S4). The detected non-cavity residues that surround the cavity region may have functional roles in membrane protein transporters.

Different MFS transporters may have diverse interaction networks of cooperative residues. We believe that the diversity of the networks occurs because evolution likely favors functional diversification of MFS transporters. Interestingly, we found that the interaction network of the detected residues in EmrD were found from only one symmetric half (where H^+^ translocation occurs); whereas, the networks of LacY and GlpT covered both symmetric halves. In EmrD, proton translocation and drug transport may occur at different sites in the central cavity [24]. EmrD has a large and flexible substrate recognition pocket that transports various chemically unrelated drug compounds; therefore, different drugs may interact with different sites of the pocket [57]. We suspect that the substrate recognition pocket of EmrD is not conserved so that functional residue detection is limited.

In summary, our integrative evolutionary analysis effectively shows that the conserved cores of evolutionarily coupled residues arose from functional constraints, providing information to characterize specific functional residues of MFS transporters. We believe this method can be applied to other proteins to narrow down the potential candidates of functional residues and to save time and reduce the cost incurred by molecular biology, biochemical, and biophysical approaches. We provide downloadable source code at our website (http://sbi.postech.ac.kr/IS/) for wide application of this method.

## Materials and Methods

### Sequence alignment

We obtained homologous sequences for LacY, GlpT, and EmrD of *Escherichia coli* and other membrane protein transporters from Swiss-Prot/TrEMBL. We used sequences 0.7∼1.4 times the query sequence length and <90% similarity to other sequences. We aligned the sequences using ClustalW [58]. We omitted columns with a gap ≥20% and completely conserved region.

### Quantification and integration of evolutionary information

To calculate the sequence conservation score of each residue in LacY, GlpT, EmrD, and other membrane protein transporters, we used ConSeq [59]. We compared McBASC [60], SCA [11], and ELSC [61] algorithms for co-evolutionary analysis. The precision-recall curves showed a comparable performance in the identification of cavity residues among the different algorithms (Figure S7). Among them, the McBASC algorithm performed slightly better than other algorithms, so we used the McBASC algorithm to calculate co-evolution scores. We derived the co-evolutionary coupling number (CN) through the following steps. First, we selected significant co-evolving residue pairs using a length-dependent threshold [62]. The number of co-evolving residue pairs is set equal to twice the protein length. Then, we counted the number of co-evolving residue pairs per residue and defined it as the CN. To correct the different score distributions, we normalized the sequence conservation score and CN by converting their scores into the corresponding percentile rank scores ranging from 0 to 1. Finally, we multiplied the normalized sequence conservation score by the CN to obtain the quantitative integration score (IS).

### Selecting central cavity residues

We used a set of cavity residues (positive set) and a set of non-cavity residues (negative set) to evaluate the performances of IS, co-evolution, and sequence conservation scores. The central cavity residues of transporters are composed of the residues involved in substrate recognition, which are located in the pathway of substrate transport; whereas, non-cavity residues include the rest of the central cavity residues [25]. To select central cavity residues, we measured the solvent accessible surface of translocation pathways of the three MFS transporter structures using VOIDOO with a 1.2 Å probe radius and default manner [63]. We also manually inspected the selected residues to eliminate residues from other small cavities that can occur in the structure. In LacY, 49 of 417 residues, 53 of 452 residues in GlpT, and 52 of 394 residues in EmrD are in the central cavity and are tabulated in Table S2, S4, and S7, respectively.

### Identification of functional residues and the construction of residue interaction networks

We investigated the functional implications of residues within the 90^th^ percentile of IS. At the 90^th^ percentile of IS, we can identify cavity residues with 5% false-positive rate, the fraction of non-cavity residues selected from the given percent cutoff. A 5% false-positive rate represents the acceptable level of selecting functionally important residues [64]. Based on the observation that most of the detected residues were positioned in the transmembrane region (Figure S8), we considered the residues of the transmembrane region for further analysis where important functions of MFS transporters occur. We designated transmembrane boundaries for the three MFS transporters using the Protein Data Bank of Transmembrane Proteins (PDBTM) [65]. We assessed the interatomic connectivity among the detected residues based on the crystal structures of MFS transporters in the Protein Data Bank (http://www.rcsb.org); PDB ID: 2CFQ for LacY, PDB ID: 1PW4 for GlpT, and PDB ID: 2GFP for EmrD. To measure interactions between residues, we used the contacts of structural units (CSU) software (http://www.weizmann.ac.il/sgedg/csu/). In a given protein structure, the CSU software provides a list of interatomic interactions and their distances by measuring the solvent-accessible surface of every atoms of two residues [66]. A van der Waals interaction was identified if the distance between any two atoms of the residues is less than the sum of their van der Waals radii plus the diameter of a solvent molecule (2.8Å). A salt bridge was identified when the distance between the donor atoms (N^ζ^ of Lys, N^ζ^, N^η1^, N^η2^ of Arg, N^δ1^, N^ε2^ of His) and the acceptor atoms (O^ε1^, O^ε2^ of Glu, O^δ1^, O^δ2^ of Asp) was less than 4.0 Å [67]. A hydrogen bond was assessed by HBPLUS [68], which measures the angle and distance of each donor-acceptor pair to find out its fitness to the geometric criteria defined by Baker and Hubbard [69].

### Likelihood ratio calculation

We used likelihood ratios to statistically evaluate how well different evolutionary features (IS, CN, co-evolution, and sequence conservation scores) could discriminate central cavity residues from non-cavity residues for each of the following percentile groups: 80%, 82%, 84%, 86%, 88%, 90%, 92%, 94%, and 96%. We obtained likelihood ratios for different evolutionary features with:
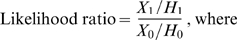
(1)X_1_ and X_0_ represent the number of central cavity and non-cavity residues selected from the given percent cutoff, respectively. H_1_ indicates the total number of central cavity residues. H_0_ is the total number of non-cavity residues. A likelihood ratio >1 indicates a reliable probability. An increasing likelihood ratio signifies the detection of more central cavity residues.

### Data collection for extensive test to identify cavity residues

We tested our method on other membrane protein transporters. We collected the membrane protein transporters whose allosteric conformational changes were characterized and cavity residues can be selected from the crystal structures. We chose 15 protein structures from the five largest families of membrane protein transporters, which include KvAP and Kv1.2 voltage-gated K^+^ channels, rhodopsin, chloride pump halorhodopsin, bacteriorhodopsin, sensory rhodopsin, archaerhodopsin, Na^+^/K^+^ ATPase, P-type Ca2^+^ ATPase, plasma membrane ATPase, and sulfate/molybdate ABC transporter. Cavity residues were selected, as described in the procedure for selecting central cavity residues in MFS transporters.

## Supporting Information

Figure S1 Sliding window plots of sequence conservation-to-fraction of central cavity residues in LacY (A), GlpT (B), and EmrD (C).
(0.09 MB PDF)

Figure S2 Likelihood ratios of IS, CN, co-evolution, and sequence conservation scores.
(0.04 MB PDF)

Figure S3 IS pattern of GlpT and EmrD.
(0.06 MB PDF)

Figure S4 Interaction networks of the high-IS residues of membrane protein transporters.
(0.08 MB PDF)

Figure S5 Precision-recall curves of four evolutionary approaches.
(0.04 MB PDF)

Figure S6 Interaction networks of the detected residues of LacY.
(0.08 MB PDF)

Figure S7 Precision-recall curves of three algorithms for co-evolutionary analysis.
(0.03 MB PDF)

Figure S8 Positions of the detected functional residues are shown with the Z-coordinates of MFS transporters (A) LacY, (B) GlpT, and (C) EmrD.
(0.09 MB PDF)

Table S1 Differences of IS, CN, and sequence conservation score between central cavity and non-cavity region.
(0.10 MB XLS)

Table S2 List of central cavity residues in lactose permease (LacY).
(0.14 MB XLS)

Table S3 Interaction network of detected residues in LacY.
(0.13 MB XLS)

Table S4 List of central cavity residues in glycerol-3-phosphate transporter (GlpT).
(0.14 MB XLS)

Table S5 Interaction network of detected residues in GlpT.
(0.13 MB XLS)

Table S6 Functional implications and experimental evidence of the detected GlpT residues.
(0.11 MB XLS)

Table S7 List of central cavity residues in multidrug transporter EmrD.
(0.13 MB XLS)

Table S8 Interaction network of detected residues in EmrD.
(0.12 MB XLS)

Table S9 Functional implications and experimental evidence of the detected EmrD residues.
(0.10 MB XLS)

Table S10 Identified functional residues of membrane protein transporters.
(0.11 MB XLS)
