# Supplementary material for: Integration of Evolutionary Features for the Identification of Functionally Important Residues in Major Facilitator Superfamily Transporters
Source: PLoS Comput Biol. 2009 Oct 2;5(10):e1000522. doi: 10.1371/journal.pcbi.1000522 (PMC2739438; doi:10.1371/journal.pcbi.1000522)
Supplement: Figure S1 — Sliding window plots of sequence conservation-to-fraction of central cavity residues in LacY (A), GlpT (B), and EmrD (C). (0.09 MB PDF) [file pcbi.1000522.s001.pdf]

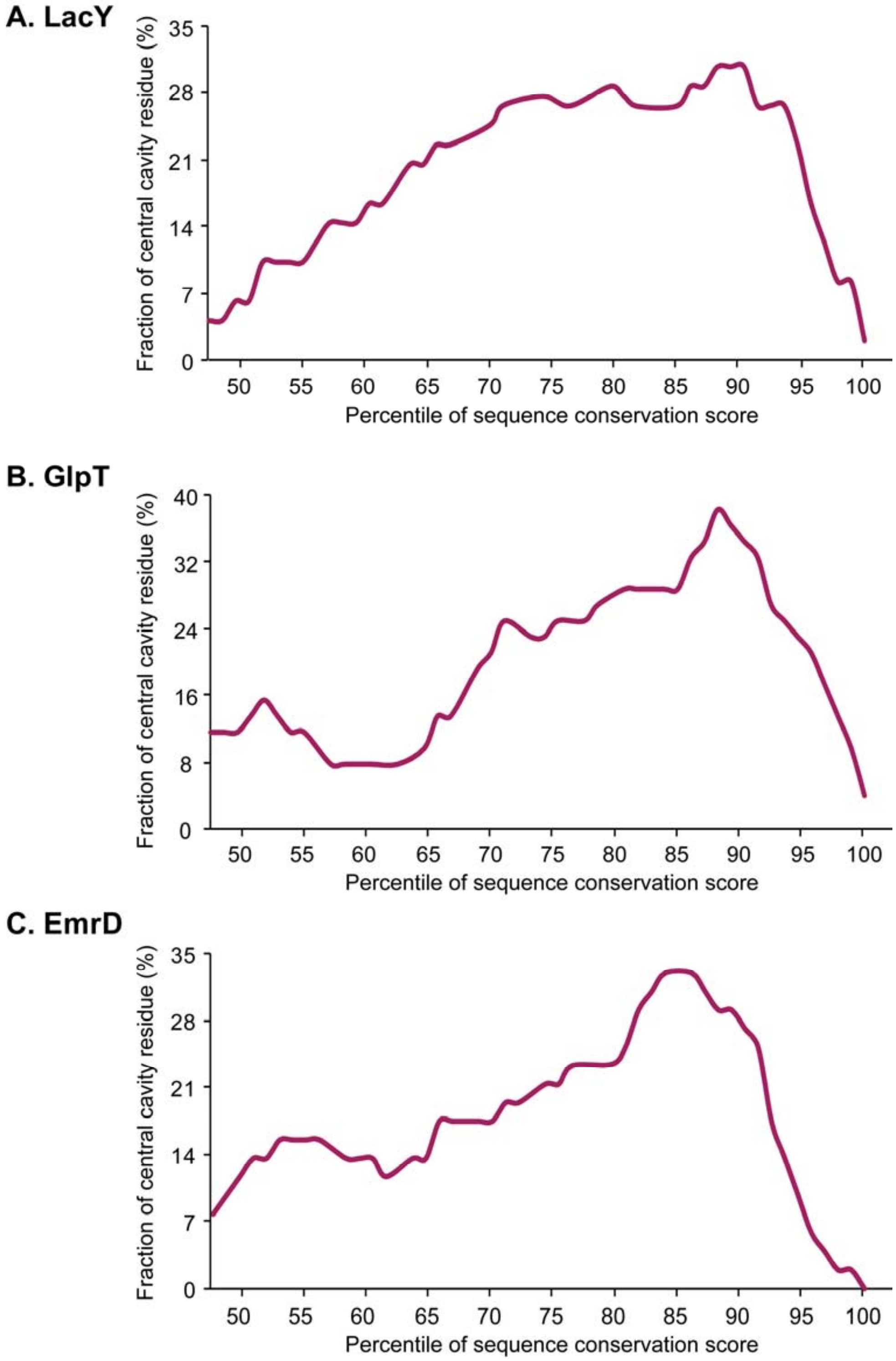

Figure S1. Sliding window plots of sequence conservation-to-fraction of central cavity residues in LacY (A), GlpT (B), and EmrD (C) with a window size 10 % and a step size 1 %.
