# Supplementary material for: Integration of Evolutionary Features for the Identification of Functionally Important Residues in Major Facilitator Superfamily Transporters
Source: PLoS Comput Biol. 2009 Oct 2;5(10):e1000522. doi: 10.1371/journal.pcbi.1000522 (PMC2739438; doi:10.1371/journal.pcbi.1000522)
Supplement: Figure S2 — Likelihood ratios of IS, CN, co-evolution, and sequence conservation scores. (0.04 MB PDF) [file pcbi.1000522.s002.pdf]

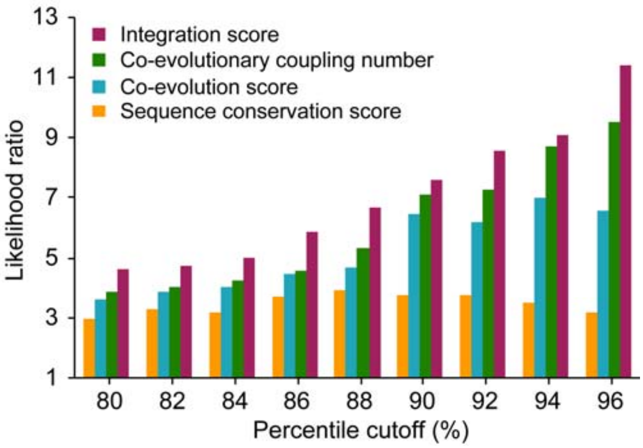

Figure S2. Likelihood ratios of IS, CN, co-evolution, and sequence conservation scores. Likelihood ratio statistically evaluate how well different evolutionary approaches (IS, CN, co-evolution, and sequence conservation score) discriminate central cavity residues from non-cavity residues for each of the following percentile groups. Red, green, blue, and yellow indicate the average likelihood ratio of IS, CN, co-evolution, and sequence conservation in the given percentile cutoff, respectively.
