# Supplementary material for: Integration of Evolutionary Features for the Identification of Functionally Important Residues in Major Facilitator Superfamily Transporters
Source: PLoS Comput Biol. 2009 Oct 2;5(10):e1000522. doi: 10.1371/journal.pcbi.1000522 (PMC2739438; doi:10.1371/journal.pcbi.1000522)
Supplement: Figure S3 — IS pattern of GlpT and EmrD. (0.06 MB PDF) [file pcbi.1000522.s003.pdf]

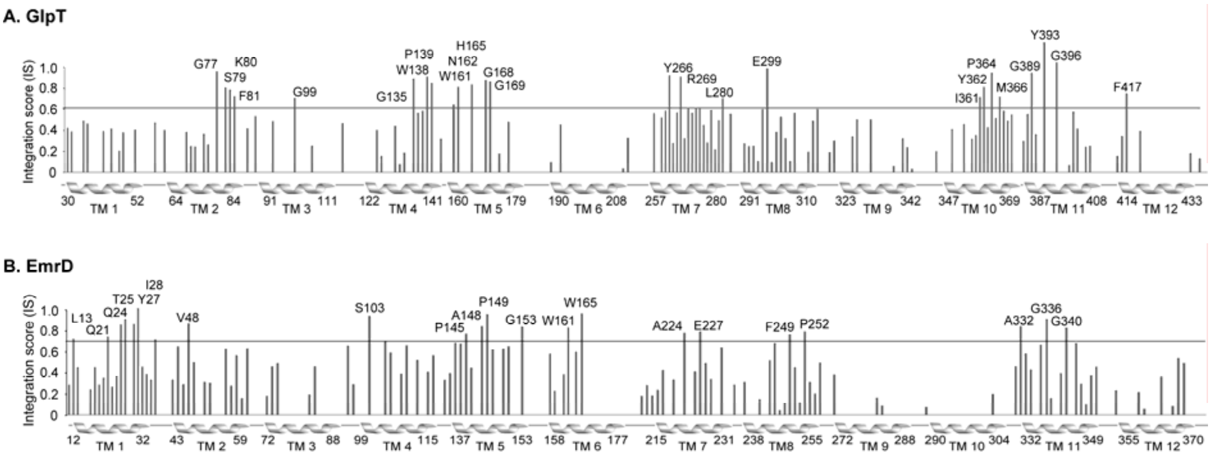

Figure S3. IS pattern of GlpT and EmrD. (A) In GlpT, 25 detected residues are labeled with residue numbers. Black line corresponds to the 90th percentile of IS. (B) In EmrD, 21 detected residues are labeled with residue numbers.
