# Supplementary material for: Integration of Evolutionary Features for the Identification of Functionally Important Residues in Major Facilitator Superfamily Transporters
Source: PLoS Comput Biol. 2009 Oct 2;5(10):e1000522. doi: 10.1371/journal.pcbi.1000522 (PMC2739438; doi:10.1371/journal.pcbi.1000522)
Supplement: Figure S4 — Interaction networks of the high-IS residues of membrane protein transporters. (0.08 MB PDF) [file pcbi.1000522.s004.pdf]

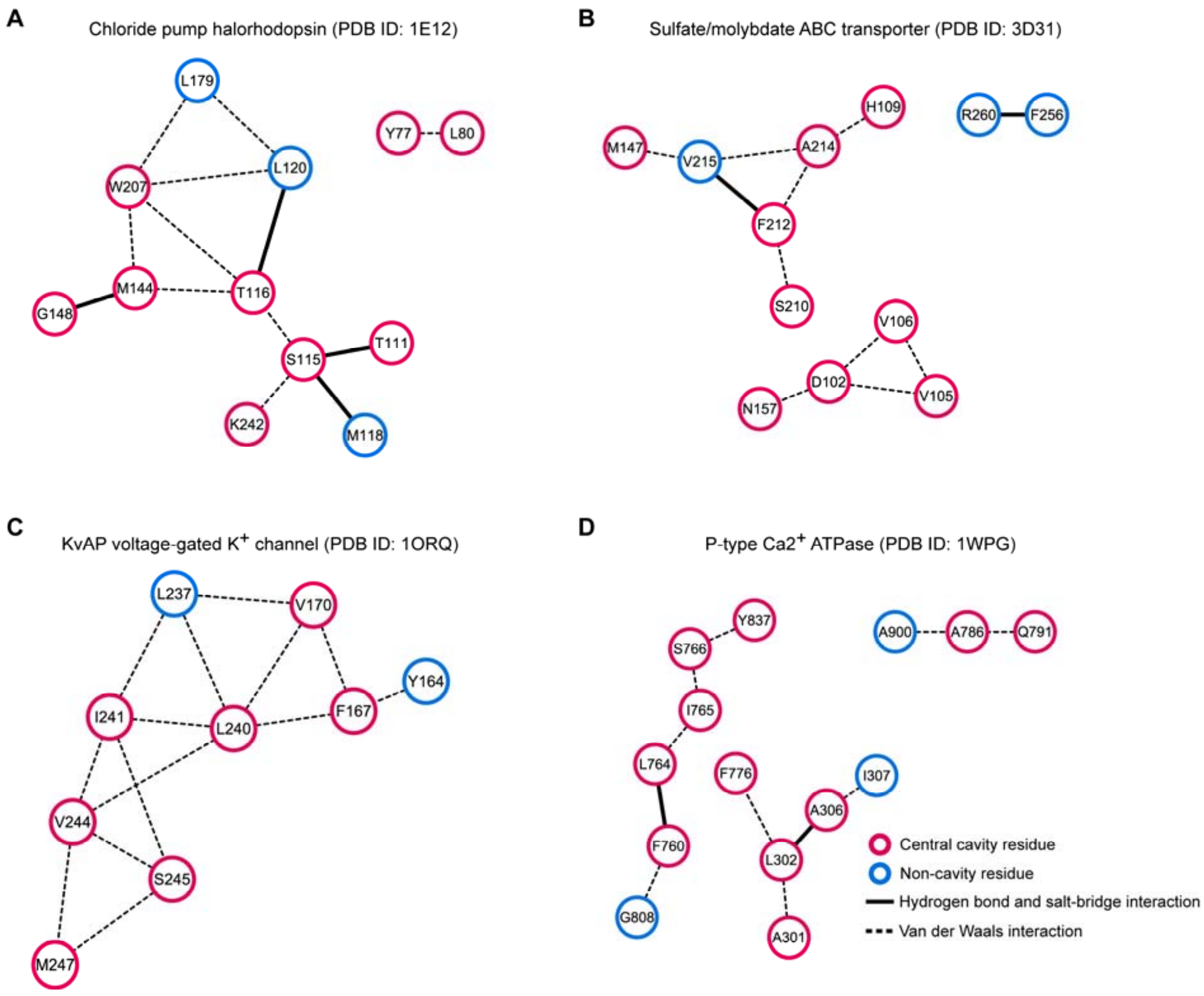

Figure S4. Interaction networks of the high-IS residues of membrane protein transporters. Red circle represents cavity residues and blue circle indicates non-cavity residues. (A) Chloride pump halorhodopsin (PDB ID: 1E12), (B) Sulfate/molybdate ABC transporter (PDB ID: 3D31), (C) KvAP voltage-gated K^+ channel (PDB ID: 1ORQ), and (D) P-type $Ca2^+$ ATPase (PDB ID: 1WPG).
