# Supplementary material for: Integration of Evolutionary Features for the Identification of Functionally Important Residues in Major Facilitator Superfamily Transporters
Source: PLoS Comput Biol. 2009 Oct 2;5(10):e1000522. doi: 10.1371/journal.pcbi.1000522 (PMC2739438; doi:10.1371/journal.pcbi.1000522)
Supplement: Figure S5 — Precision-recall curves of four evolutionary approaches. (0.04 MB PDF) [file pcbi.1000522.s005.pdf]

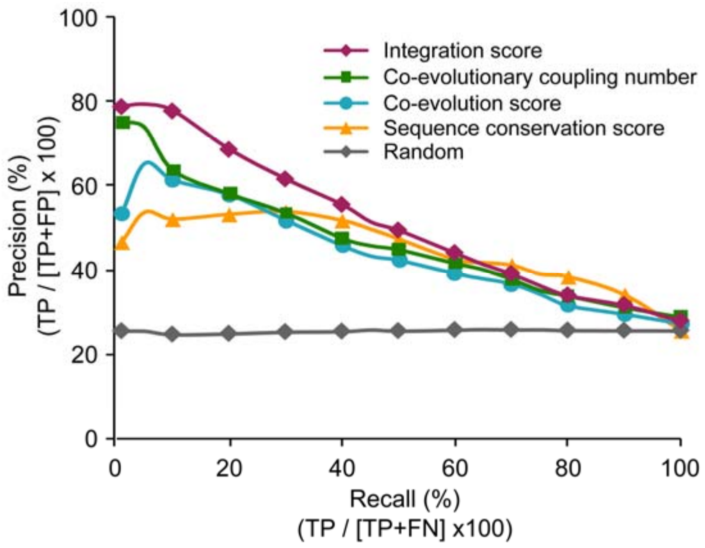

Figure S5. Precision-recall curves of four evolutionary approaches. Precision and recall were derived from cavity residues (positive set) and non-cavity residues (negative set) of 15 membrane protein transporters. Red, green, blue, and yellow dots represent the average precisions of each evolutionary approach in the given recall.
