# Supplementary material for: Integration of Evolutionary Features for the Identification of Functionally Important Residues in Major Facilitator Superfamily Transporters
Source: PLoS Comput Biol. 2009 Oct 2;5(10):e1000522. doi: 10.1371/journal.pcbi.1000522 (PMC2739438; doi:10.1371/journal.pcbi.1000522)
Supplement: Figure S6 — Interaction networks of the detected residues of LacY. (0.08 MB PDF) [file pcbi.1000522.s006.pdf]

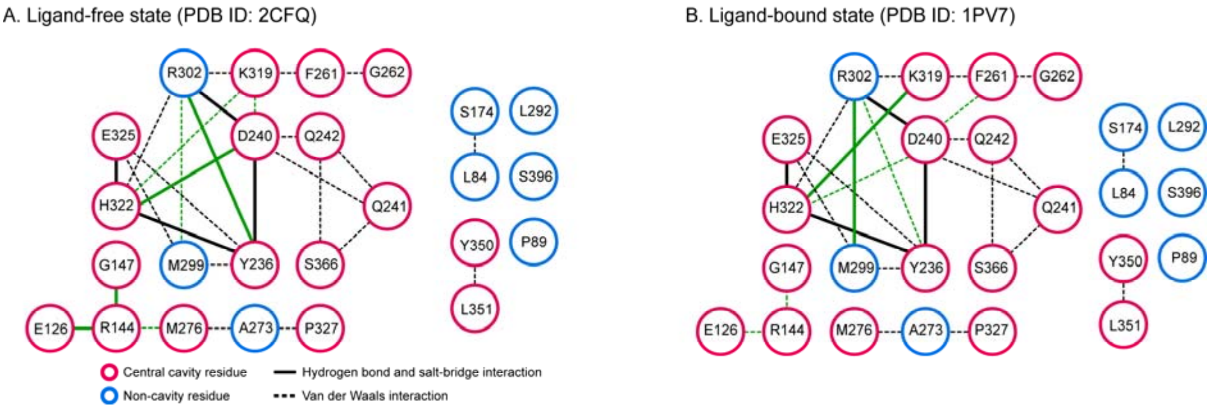

Figure S6. Interaction networks of the detected residues of LacY. Ligand-free (PDB ID: 2CFQ; left) and ligand-bound structures of LacY (PDB ID: 1PV6; right) are shown. The changed interactions of residues between two structures are shown in green. Bold lines indicate potential hydrogen bonds or salt bridges, while dashed lines represent van deer Waals interactions.
