# Supplementary material for: Integration of Evolutionary Features for the Identification of Functionally Important Residues in Major Facilitator Superfamily Transporters
Source: PLoS Comput Biol. 2009 Oct 2;5(10):e1000522. doi: 10.1371/journal.pcbi.1000522 (PMC2739438; doi:10.1371/journal.pcbi.1000522)
Supplement: Figure S7 — Precision-recall curves of three algorithms for co-evolutionary analysis. (0.03 MB PDF) [file pcbi.1000522.s007.pdf]

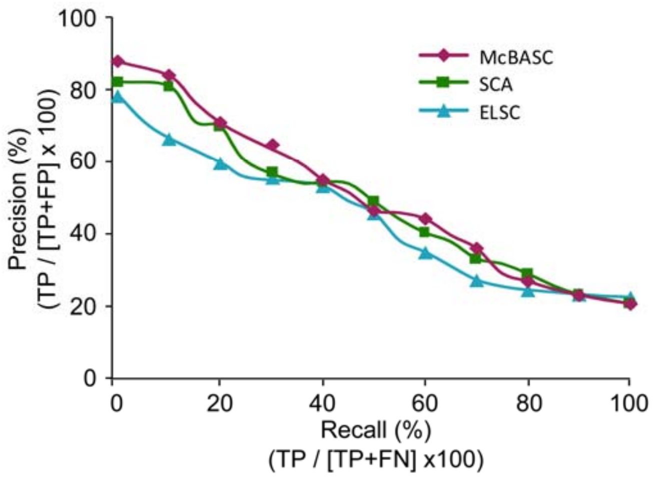

Figure S7. Precision-recall curves of three algorithms for co-evolutionary analysis. Precision-recall curves of McBASC, SCA, and ELSC algorithms were analyzed for the comparison of three different co-evolutionary methods. Red, green, and blue dots represent the average precisions of each algorithm in the given recall.
