# Supplementary material for: Integration of Evolutionary Features for the Identification of Functionally Important Residues in Major Facilitator Superfamily Transporters
Source: PLoS Comput Biol. 2009 Oct 2;5(10):e1000522. doi: 10.1371/journal.pcbi.1000522 (PMC2739438; doi:10.1371/journal.pcbi.1000522)
Supplement: Figure S8 — Positions of the detected functional residues are shown with the Z-coordinates of MFS transporters (A) LacY, (B) GlpT, and (C) EmrD. (0.09 MB PDF) [file pcbi.1000522.s008.pdf]

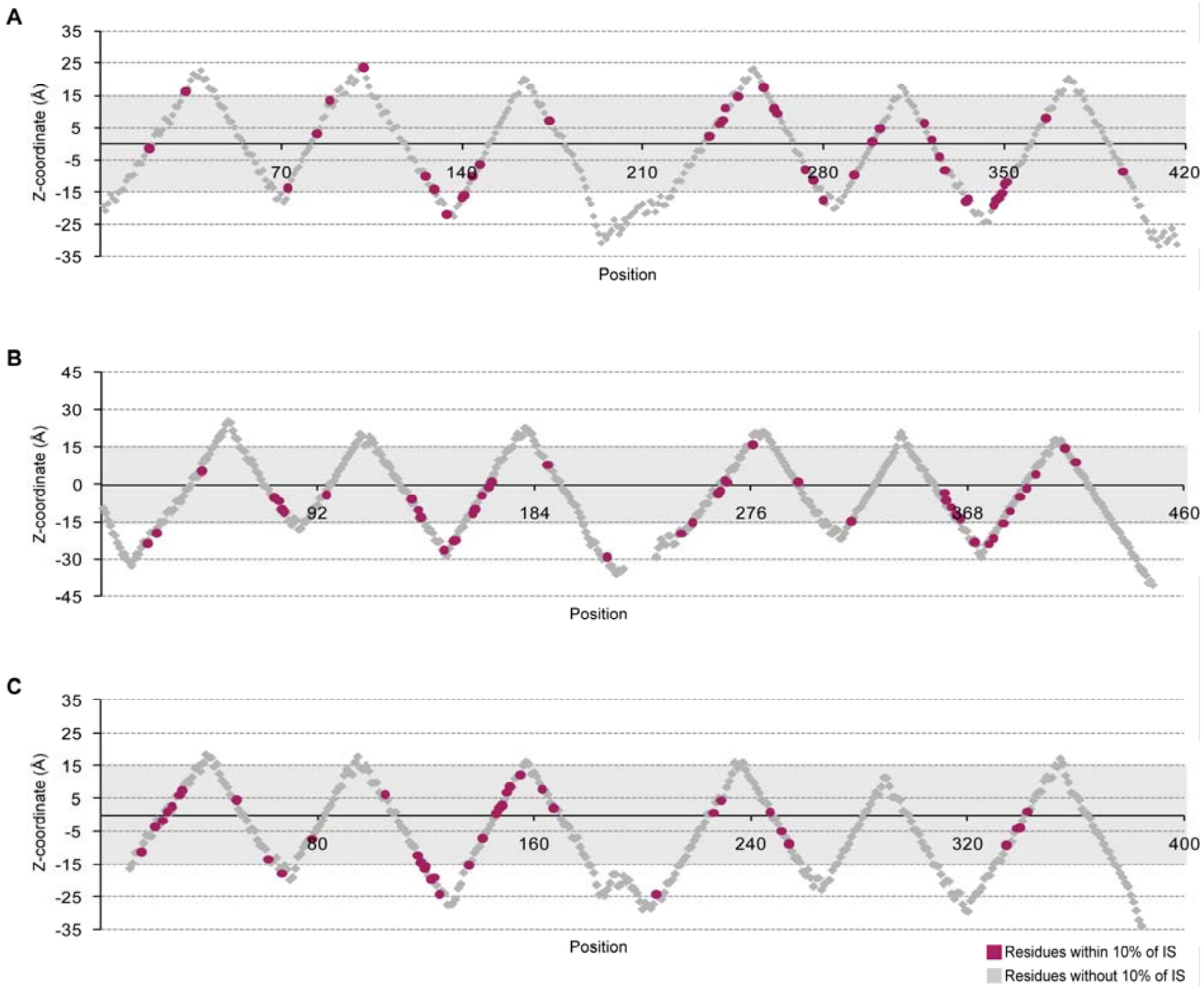

Figure S8. Positions of the detected functional residues are shown with the Z-coordinates of MFS transporters (A) LacY, (B) GlpT, and (C) EmrD. Red squares indicate detected residues within the top 10% of IS. Shaded box on Z-coordinates represents the transmembrane region (-15A ~ +15 A from membrane center). Negative and positive values of Z-coordinate indicate the orientation of cytoplasmic and periplasmic sides, respectively. Most of the detected residues are found in the transmembrane region or near to the transmembrane region.
